# Development of an Assay for the Identification of Receptor Binding Proteins from Bacteriophages

**DOI:** 10.3390/v8010017

**Published:** 2016-01-11

**Authors:** David J. Simpson, Jessica C. Sacher, Christine M. Szymanski

**Affiliations:** Alberta Glycomics Centre and Department of Biological Sciences, University of Alberta, Edmonton, AB T6G 2E9, Canada; djsimpso@ualberta.ca (D.J.S.); sacher@ualberta.ca (J.C.S.)

**Keywords:** bacteriophage receptor binding protein, tailspike protein, expression library screen

## Abstract

Recently, a large number of new technologies have been developed that exploit the unique properties of bacteriophage receptor binding proteins (RBPs). These include their use in diagnostic applications that selectively capture bacteria and as therapeutics that reduce bacterial colonization *in vivo*. RBPs exhibit comparable, and in many cases superior, stability, receptor specificity, and affinity to other carbohydrate binding proteins such as antibodies or lectins. In order to further exploit the use of RBPs, we have developed an assay for discovering RBPs using phage genome expression libraries and protein screens to identify binding partners that recognize the host bacterium. When phage P22 was screened using this assay, Gp9 was the only RBP discovered, confirming previous predictions that this is the sole RBP encoded by this phage. We then examined the *Escherichia coli* O157:H7 typing phage 1 in our assay and identified a previously undescribed RBP. This general approach has the potential to assist in the identification of RBPs from other bacteriophages.

## 1. Introduction

Bacteriophage receptor binding proteins (RBPs) have recently been developed into a number of tools that make use of their high specificity and robustness [1]. These technologies include diagnostics involving RBPs bound to surfaces [2], or to beads [3,4], for the selective capture of bacteria. Moreover, RBPs have also been employed as therapeutics that reduce specific bacterial colonization *in vivo*, either alone [5] or as part of a larger molecular machine [6]. As diagnostics, RBPs offer several advantages over other technologies, such as antibodies, including greater stability, ligand specificity, and affinity even against carbohydrate epitopes which are not typically recognized effectively by most antibodies [7,8]. The importance of developing technologies for the rapid identification of pathogens cannot be understated, as food contaminated with bacterial pathogens presents both a public health concern and a serious financial liability for producers. Also, rapid methods of bacterial detection can help surmount challenges in diagnosing bacterial agents of disease prior to progression and/or spread of the infection. RBPs therefore represent a highly versatile, effective and much-needed technology for bacterial pathogen detection.

Phages recognize their hosts through RBP binding to a specific receptor on the host cell surface. RBPs are also responsible for properly orienting the phage onto the host cell, which must occur prior to a successful infection [9]. These proteins can be called tailspikes, tail fibres or spike proteins. Due to their diversity in host-binding specificity, RBP genes often cannot be recognized in a sequenced genome based solely on homology with already characterized RBPs [10], since even if the phage RBPs share structural homology, they do not tend to share sequence homology [11,12]. Known phage RBPs have been shown to be very stable proteins, displaying high resistance to proteases and detergents. These properties are inherent to phage RBPs, presumably since these proteins have evolved to be functional in harsh native environments such as the intestinal tract. On a molecular level, this stability is generally attributed to the fact that RBPs are often trimers rich in β-structures that are intertwined in a β-helical architecture. The phage P22 trimeric tailspike, Gp9, serves as a classic example of RBP spatial arrangement [1]. Overall, the high stability, specificity, and ease of recombinant overexpression make RBPs excellent alternatives to antibodies and ideal tools for the development of new diagnostic technologies.

In addition, RBPs are not limited to phages, but can also be found as components of phage-like molecular machines such as pyocins [13] and gene transfer agents (GTAs) [14]. Pyocins, also known as headless phages, bind to a target cell and create a channel across the membrane(s), disrupting the transmembrane potential and killing the cell [15]. These machines are currently being exploited as antibacterial agents and have had their target range altered by substituting different RBPs with their host recognition moieties, giving known pyocins novel target ranges [16]. GTAs are non-replicative phage-like particles that package portions of the bacterial chromosome and inject that DNA into a neighboring target cell [14]. Both of these machines rely on their RBPs for binding to and identification of their host, and thus present other natural reservoirs for proteins of this class.

Considerable research has been done on RBPs, especially the P22 RBP, which has been used as a model for understanding protein folding and phage infection [9]. However, although lambda phage has been shown to be a useful tool for annotating phage genomes, to date there is no rapid method for identifying phage RBPs, even within sequenced genomes. This is a result of the fact that RBPs are difficult to identify based on homology, since RBPs each bind to distinct receptors. As well, phage DNA has been shown to be challenging to sequence, likely as a result of hyper-modification of the phage DNA bases [17], further contributing to challenges associated with RBP identification. In order to overcome these difficulties, we developed a method to rapidly identify RBPs in the absence of sequence information. We generated *E. coli* gene expression libraries expressing randomly sheared fragments of phage DNA and then screened these libraries for colonies producing a gene product able to bind to the host organism. We tested this technique with two phages: *Salmonella enterica* serovar Typhimurium phage P22, which belongs to the *Podoviridae* family, and *E. coli* O157:H7 typing phage 1, belonging to the *Myoviridae* family [18,19]. In both cases, the tailspike or putative tail fibre was successfully identified using the screen and all identified genome fragments contained coding regions for putative RBPs. Together these results demonstrate the utility of an assay for accurate RBP identification from phages belonging to two of the three major families of tailed phages.

## 2. Materials and Methods

### 2.1. Strains and Phages

The bacteriophage P22 and its propagating strain *S.* Typhimurium ATCC 19585 were obtained from the American Type Culture Collection. The *E. coli* O157:H7 typing phage 1 and its propagating strain C-8299-83 were obtained from the Félix d’Hérelle Centre (Université Laval, Quebec, QC, Canada).

### 2.2. Construction of Gene Expression Libraries

Phage DNA was extracted using a Phage DNA Isolation Kit (Norgen Biotek Corp, Thorold, ON, Canada) according to the manufacturer’s instructions. The phage DNA was then nebulized using a nebulizer kit (Invitrogen, Waltham, MA, USA). Briefly, 10 µg DNA was diluted in 1 mL of shearing buffer (10% glycerol, 10 mM Tris, 1 mM EDTA, pH 8) and nebulized under 10 psi of nitrogen gas for 30 s. The sheared DNA was precipitated overnight at −20 °C in isopropanol, 40 μg/mL glycogen and 0.3 M sodium acetate (pH 4.8) and then blunt-end repaired using T4 DNA polymerase (Thermo Scientific, Waltham, MA, USA) according to the manufacturer’s instructions. The DNA fragments were precipitated again in isopropanol and then ligated into the dephosphorylated (FastAP Thermo Scientific) *Eco*RV site of pET30a using T4 DNA ligase (NEB). Next, 20 ng of pET30a was ligated with 50, 100 or 200 ng of sheared phage DNA in a 20 µL reaction volume overnight at 16 °C, and the ligation mixture was transformed into chemically competent Top10 cells (Invitrogen). The resulting colonies were then screened for the presence of inserted DNA into pET30a. If at least 2/10 verified plasmids contained inserts between 1 and 3 kB, the entire library was plated out for single colonies, pooled together and plasmid DNA was extracted using a plasmid mini prep kit (Thermo Scientific). The library was then transformed into *E. coli* BL21(DE3) cells.

### 2.3. Screen for Identification of Receptor Binding Protein-Encoding Genes

**Figure 1:**
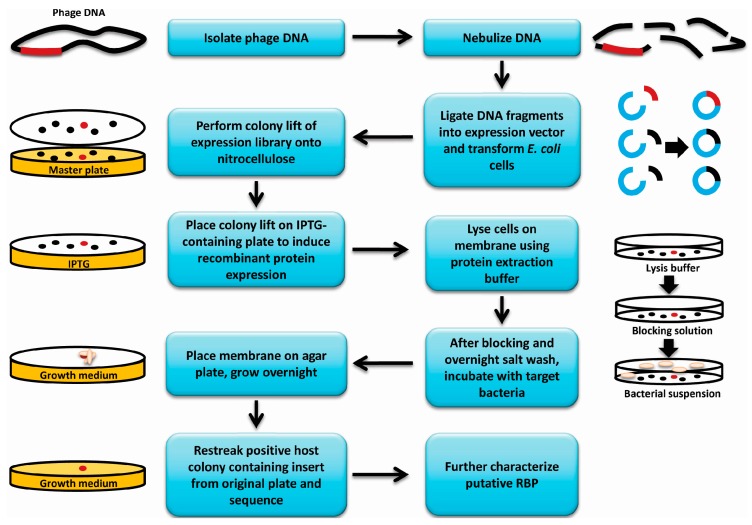
Overview of receptor binding protein (RBP) discovery assay. A flowchart and graphical representation summarizing the methodology used to discover new phage receptor binding proteins. Red dots represent an *E. coli* colony expressing an RBP gene among a library of colonies expressing other fragments of the genome (black dots). Red gene fragments represent an RBP gene, while black gene fragments represent other fragments of the genome. Blue fragments represent vector DNA, and beige ovals represent bacterial cells incubated with the colony lift membrane.

*E. coli* BL21(DE3) cells containing the phage genomic expression library described above were plated for isolated colonies (150 to 200 colonies per plate) on Lysogeny Broth (LB) containing 25 µg/mL kanamycin (Figure 1). A nitrocellulose membrane cut to fit each plate was placed on top of the colonies, gently pressed down and removed after 5–10 s. Membranes were then placed colony side up on LB (25 µg/mL kanamycin + 0.4 mM IPTG) agar plates and incubated overnight at 30 °C for induction of protein expression. The colony lifts were then incubated colony side up for 1 h at room temperature on top of a similarly sized Whatman filter paper that was pre-saturated with bacterial protein extraction reagent (B-PER, Thermo Scientific) containing 1× protease inhibitor cocktail (Roche Laval, Laval, QC, Canada), DNAse I (1 U/mL) (Thermo Scientific) and lysozyme (500 µg/mL) (Sigma Canada, Oakville, ON, Canada). The colony lifts were blocked for 1 h in 5% skim milk (Difco, Mississauga, ON, Canada), washed in Phosphate buffered Saline pH 7.4 with 0.5% Tween 20 (PBST), and remnants of lysed colonies were then gently wiped off using a Kimwipe. The membranes were then exposed to ultraviolet light for 15 min to kill any remaining intact *E. coli* cells. The lifts were incubated overnight in 500 mM NaCl at 4 °C and then incubated in a suspension of the host organism at 10^8^ colony forming units (CFU)/mL in blocking solution for 30 min at room temperature with gentle shaking. The membranes were then washed 3 × 10 min in PBST, dried by dabbing with a paper towel and then placed colony side up on LB agar plates. Plates were incubated overnight at room temperature to allow bound bacterial cells to grow into visible colonies on the membrane. The colonies that appeared on the membranes were matched to their corresponding colonies on the master plates containing the original *E. coli* expression library and the corresponding colonies were patched to a new plate. Patched colonies were used in a subsequent colony lift and carried through a second round of the assay to confirm the results. The genomic expression library colonies that demonstrated a positive result in the second round were grown up, frozen down and the inserts in the plasmids were sequenced.

### 2.4. Imaging of Colony Lifts with Green Fluorescence Protein (GFP) Expressing S. Typhimurium

**Figure 2:**
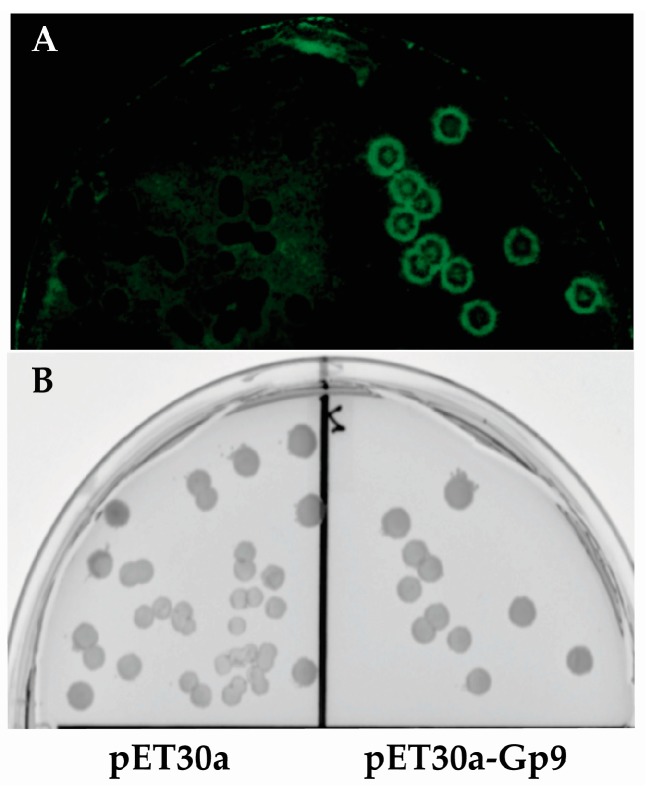
Image of colony lift. (**A**) Fluorescent image of Green Fluorescence Protein (GFP) expressing *S.* Typhimurium growing on a nitrocellulose membrane. (**B**) The corresponding master plate of the colony lift showing *E. coli* colonies from the empty vector control (pET30a) on the left and *E. coli* colonies expressing the positive insert (16155–19050) from the assay on the right.

Since it is difficult to photograph bacterial colonies growing on nitrocellulose membranes, colonies of *E. coli* BL21(DE3) containing either the empty vector (pET30a) or plasmid expressing the Gp9 insert 16155–19050 that was identified in this study (pET30a-Gp9) were subjected to the assay conditions and then probed with *S*. *Typhimurium* expressing GFP (pWM1007 [20]) to generate a visual image of the assay (Figure 2). After overnight growth, the membranes were imaged with a FujiFilm FLA-5000 system (Fuji Photo Film Co., Ltd., Tokyo, Japan) using the 473 nm laser at 400 V for excitation and LPB (Y510) filter for emission.

### 2.5. Protein Modelling

All putative RBPs were sent to the Phyre 2 server for protein modeling available online) [21]. Table 1 shows the top result for each of the genes discovered in the assay and all results with a confidence greater than 90%.

**Table 1:** Phyre analysis of the putative ECTP1 RBPs isolated from the assay.

Protein	Phyre Prediction	Identity	Confidence
ECTP1_00144	Baseplate structural protein Gp10 (T4 phage)	19%	99.2%
	Receptor binding tip Gp37 (T4)	27%	99.1%
ECTP1_00145	Baseplate structural protein Gp10 (T4)	43%	100%
	Receptor binding tip Gp37 (T4)	35%	99.9%
	Short tail fibre Gp12 (T4)	16%	98.9%
ECTP1_00146	Restriction endonuclease	100%	28%
ECTP1_00147	Restriction endonuclease	20%	56.3%
ECTP1_00148	Baseplate structural protein Gp6	12%	94.2%

## 3. Results

**Figure 3:**
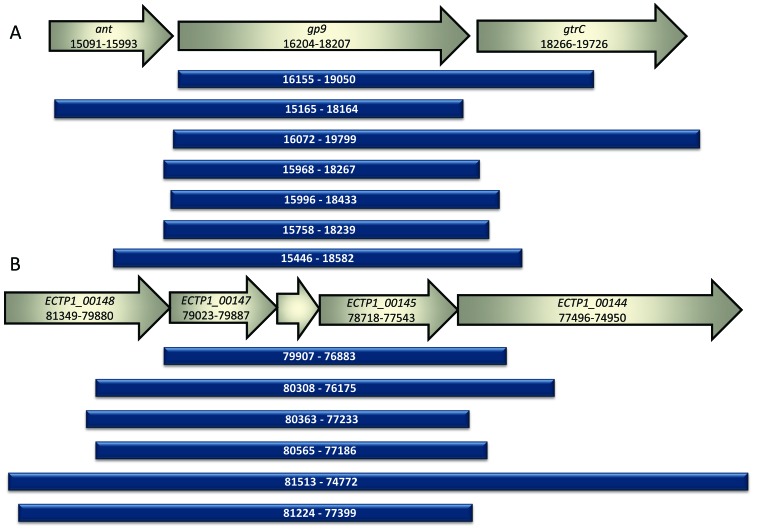
Alignment of sequenced fragments from positive colonies compared to GenBank annotated sequences. (**A**) The inserts identified in this study following screening of the P22 phage genomic library are shown alongside the corresponding portion of the phage P22 genome. *Ant* encodes the antirepressor associated with the prophage switch from lysogenic to lytic mode, *gp9* encodes the known P22 phage tailspike protein and *gtrC* encodes the *O*-antigen conversion glucosyltransferase. All inserts discovered using the assay contained *gp9.* The numbers correspond to the location in the P22 phage genome BK000583.1; (**B**) The inserts identified in this study following screening of the *E. coli* O157 typing phage 1 library are shown alongside the corresponding portion of the phage 1 genome. All inserts contained the unknown genes *ECTP1_00145*, *ECTP1_00146* and *ECTP1_00147*. *ECTP_00145* was predicted to be a tail fibre by protein modelling. *ECTP1_00146* is in between *ECTP1_00147* and *ECTP1_00145* and is unmarked in the figure. The numbers correspond to the location in the phage ECTP1 genome KP869100.1.

The *S.* Typhimurium phage P22 library produced seven unique gene fragments that bound to *S*. Typhimurium. All fragments contained an intact copy of the known tailspike gene, *gp9*. Approximately 5700 colonies were screened, with 70% containing inserts. Eleven positive colonies were obtained, all of which contained the known tailspike gene *gp9*, and seven of these contained unique inserts. All inserts were in the correct orientation for expression from the vector promoter (Figure 3A).

The *E. coli* O157:H7 typing phage 1 library produced six unique gene fragments that bound to *E. coli* O157. Approximately 4000 colonies were screened, with 30% containing inserts. Eight colonies showed positive binding, with six of these containing unique inserts. All inserts were in the correct orientation for expression from the vector promoter (Figure 3B). The observed difference in the percentage of vectors containing inserts is likely due to constructing the P22 library using DNA that had undergone extra cleaning steps.

**Figure 4:**
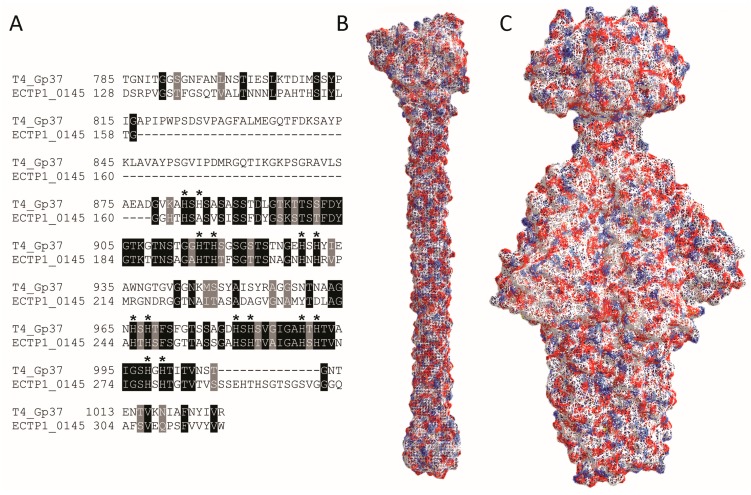
Alignment and structure of RBPs detected in this study. (**A**) BlastP alignment of phage T4 Gp37 receptor binding tip (amino acids 785–1026 from 1026 in the full length protein), and the corresponding homologous region of ECTP1_00145 (amino acids 128–317 from 391), showing conserved amino acids in light grey and identical amino acids in dark grey. The dashes represent gaps in the alignment. Histidines involved in iron coordination are marked with asterisks; (**B**) Protein structure of the T4 Gp37 receptor binding tip trimer (785–1026, PDB ID: 2XGF) [22]; (**C**) Protein structure of the P22 Gp9 trimer (PDB ID: 2XC1) [23]. The red and blue colours indicate negative and positive residues while the white surfaces show hydrophobic residues. Alignment and structure of RBPs detected in this study. (**A**) BlastP alignment of phage T4 Gp37 receptor binding tip (amino acids 785–1026 from 1026 in the full length protein), and the corresponding homologous region of ECTP1_00145 (amino acids 128–317 from 391), showing conserved amino acids in light grey and identical amino acids in dark grey. The dashes represent gaps in the alignment. Histidines involved in iron coordination are marked with asterisks; (**B**) Protein structure of the T4 Gp37 receptor binding tip trimer (785–1026, PDB ID: 2XGF) [22]; (**C**) Protein structure of the P22 Gp9 trimer (PDB ID: 2XC1) [23]. The red and blue colours indicate negative and positive residues while the white surfaces show hydrophobic residues.

All inserts contained the genes *ECTP1_00145-00147*. *ECTP1_00146* and *ECTP1_00147* were both predicted with low confidence to be restriction endonucleases. *ECTP1_00145*, which when translated and submitted to Phyre2 analysis was modeled to have homology to the Gp37 tail fibre from the T4 phage and to the baseplate protein Gp10 from T4. ECTP1_00144 and ECTP1_00148 were also submitted to the Phyre server, but while ECTP1_00144 was also predicted to be a tail fibre, it was only isolated in one insert. Even then, this protein was isolated together with ECTP1_00145, so it is unlikely that ECTP1_00144 is the RBP responsible for *E. coli* binding in this case (Table 1). Since Gp37 is the known RBP for T4, we further compared Gp37 and ECTP1_00145 (Figure 4) and found limited homology between the two. BlastP analysis showed 36% sequence identity between Gp37 and ECTP1_00145 over 23% sequence coverage, with an *E*-value of 6 × 10^−29^ (Figure 4A). Interestingly, most of the homology was at the C-terminal end of the proteins, the region known to be involved in receptor binding in Gp37. However, the Gp37 domain responsible for binding, between amino acids 931 and 966 [22], was less conserved with ECTP1_00145. The difference in this particular region may be indicating that the two proteins have different receptors. Interestingly, all but one of the 14 histidine residues found in this region, which form pairs needed for iron coordination, were conserved in ECTP1_00145 (indicated by asterisks in Figure 4A). Although only the 24.6 kDa binding tip of the full 109.2 kDa Gp37 protein has been crystallized, other putative Gp37-like tail fibres have been described that more closely resemble ECTP1_00145 in size and sequence (Table S1). As expected, there was even less homology between T4 Gp37 and P22 Gp9, with 35% identity over 19% sequence coverage and an *E*-value of 0.12. These differences are further reflected in the crystal structures of the proteins (Figure 4B,C). This lack of homology between the two proteins used in this assay demonstrates the capacity of the assay to identify very different RBPs, in this case a tailspike and a tail fibre that are encoded by two separate families of tailed phages.

## 4. Discussion

Receptor binding proteins are the components used by phages for host recognition and attachment. Previously, we along with other groups have shown that there are several applications for RBPs, both as therapeutics and as diagnostics [1,5]. However, in order to advance the usage of these technologies, a method for the timely identification of RBPs is necessary. This research describes a method for the discovery of RBPs from phages that does not depend on sequencing or bioinformatic comparison. Briefly, phage DNA is extracted, fragmented, ligated into an expression vector, transformed into *E. coli* and then transferred to a nitrocellulose membrane where recombinant proteins are overexpressed. The membrane is then probed for binding to the phage host bacterium.

This assay allows for the discovery of RBPs based on their ability to bind to a host bacterium, and thus represents an improvement over previous methods for identifying RBPs based on homology to already known RBPs or on genome synteny, since the latter methods prohibit the discovery of truly novel and unrelated RBPs. Phenotypic screens have been used in the past to discover phage proteins, including discovery of phage lysins from both isolated phages and from metagenomic samples [24]. It may eventually be possible to utilize DNA from an environmental sample to search for RBPs that originate from phages that cannot be propagated under laboratory conditions, which would help elucidate the characteristics of these phages. However, we acknowledge that the current form of this assay is limited by the fact that the target bacteria must be culturable on agar plates and that if a chaperone is necessary for RBP folding, that chaperone must be located in close proximity to the RBP in the phage genome. To circumvent the need to culture the target organism, future studies could use this method in combination with other detection methods, such as antibodies, to distinguish host bacteria bound to the nitrocellulose membranes. Interestingly, we noticed that all of the inserts discovered in the assay had the same orientation in the vector, suggesting that they were utilizing the vector promoter. However, protein expression most likely involved the native ribosome binding sites of the individual genes, as opposed to that of the vector, since the inserts were found to be translated in different frames.

To date, we have not utilized this assay for species beyond the *Enterobactericae*, but due to the wealth of proteins from other species that have been expressed in *E. coli*, which includes RBPs [25], it is likely that this assay will be amenable to RBP discovery from phages targeting other species. For example, *E. coli* BL21(DE3) has been used in the past for over-expression of Gram-positive phage tailspikes [26]. It is possible that a *Bacillus subtilis* expression system might facilitate the identification of RBPs from Gram-positive phages, but this has yet to be assessed. An *in vitro* expression system is another alternative technology that may produce superior results with some phages, such as those whose gene products are not readily expressed in *E. coli.*

This assay may also have utility beyond identifying phage RBPs. For instance, both GTAs and pyocins have RBPs, which could be detected by this assay if a library of GTA or pyocin DNA were generated. This would provide both a novel source of bacterial binding proteins and a means of better characterizing these molecular machines. Relatedly, RBPs identified in this assay could be used to alter the targets of pyocins or of whole phages.

RBPs are some of the most biotechnologically relevant proteins encoded in phage genomes, as they have evolved to specifically and effectively bind their bacterial hosts. As well, RBPs represent the most dynamic proteins encoded by most phages, as constant evolutionary arms race dynamics between phages and bacteria over time lead to a strong selection for new variants [27]. RBPs encompass a considerable diversity of binding targets, making them attractive in commercial exploitation and important for understanding the lifecycle of any phage. Over the last decade, phage research has exhibited a resurged interest, especially in the area of phage therapy. However, the fact still remains that phages, and particularly the bacterial receptors they recognize, need to be well characterized and carefully chosen in order to ensure the success of phage therapy strategies. Therefore, rapid RBP identification and careful characterization of RBP-receptor interactions is a logical and promising avenue to develop in order to help move this field forward.

## Supplementary Materials

The following are available online at www.mdpi.com/1999-4915/8/1/17/s1, Table S1: Blastx analysis of ECTP1_00145.
